# Application of RNA silencing to plant disease resistance

**DOI:** 10.1186/1758-907X-3-5

**Published:** 2012-05-31

**Authors:** Cheng-Guo Duan, Chun-Han Wang, Hui-Shan Guo

**Affiliations:** 1 State Key Laboratory of Plant Genomics and National Center for Plant Gene Research, Institute of Microbiology, Chinese Academy of Sciences, Beijing, 100101, China; 2 Present address: Department of Horticulture & Landscape Architecture, Purdue University, West Lafayette, IN, 47906, USA

**Keywords:** RNA silencing, Resistance, Virus, Viroid, PTGS, TGS, HIGS

## Abstract

To reduce the losses caused by plant pathogens, plant biologists have adopted numerous methods to engineer resistant plants. Among them, RNA silencing-based resistance has been a powerful tool that has been used to engineer resistant crops during the last two decades. Based on this mechanism, diverse approaches were developed. In this review, we focus on the application of RNA silencing to produce plants that are resistant to plant viruses such as RNA and DNA viruses, viroids, insects, and the recent expansion to fungal pathogens.

## Review

## Introduction

Plant pathogens are the cause of many plant diseases and result in substantial damage to crop production. In the past, conventional methods have been used to battle pathogen infections in plants, including cross-protection and utilization of natural resistance in plants. The pioneering work on coat protein (CP)-mediated resistance to tobacco mosaic virus (TMV) by the Beachy lab in 1986 introduced the concept of pathogen-derived resistance (PDR) [1], and multiple strategies were rapidly developed to engineer resistant plants [2,3]. These strategies are classified into two groups based on the functional molecules: protein- and RNA-mediated resistance. While the mechanisms of protein-mediated resistance are still unclear, the RNA-mediated mechanism, that is, the RNA silencing pathway, has become a powerful tool for engineering resistant plants.

RNA silencing, referred to as gene quelling in fungi and RNA interference (RNAi) in animals, is a conserved regulatory mechanism of gene expression that has been widely characterized in eukaryotic organisms. RNA silencing is a nucleotide sequence-specific process that induces mRNA degradation or translation inhibition at the post-transcriptional level (named PTGS in plants) or epigenetic modification at the transcriptional level, depended on RNA-directed DNA methylation (a process named RdDM in plants). The RNA silencing pathway is composed of a series of components: a dsRNA trigger; a processor called Dicer or a Dicer-like (DCL) protein; the processor product, small RNAs (siRNAs or miRNAs) of 21 to 24 nt in length; an effector complex called RISC in which the Argonaute (AGO) protein is the key player. siRNAs-guided AGO-cleaved target RNA may be recognized by RNA dependent RNA polymerase (RDR), which amplifies the dsRNA; and Suppressor of Gene silencing (SGS), which stabilizes the dsRNA substrate for DCLs to produce secondary siRNAs and reinforce the RNA silencing process [4].

Besides the regulatory roles in plant development, the siRNA-mediated RNA silencing also functions as a natural antiviral defense mechanism, a process named virus-induced gene silencing (VIGS) [5,6]. Host RNA silencing machinery targets and processes the virus-derived dsRNA, which is derived from pathogen replication or in a host RDR-dependent manner, into vsiRNAs (virus-derived siRNAs). The vsiRNAs are then recruited to host RISC complexes, which targets and inhibits gene expression and protein translation in the viral genome. To counteract the defense mechanism, many viruses encode a protein called viral suppressor of RNA silencing (VSR), which have been identified from diverse plant viruses [6-8]. Recently, suppressor of RNA silencing was also identified in some bacteria (BSRs) [9]. VSRs and BSRs may function in suppression of RNA silencing in different steps, either by binding siRNA duplex, or by directly interacting with key components in RNA silencing. Some of them may combine various functions to realize a multilevel suppression [10].

Based on the siRNAs-mediated RNA silencing (RNAi) mechanism, transgenic plants were designed to trigger RNA silencing by targeting pathogen genomes. Diverse targeting approaches have been developed based on the difference in precursor RNA for siRNA production, including sense/antisense RNA, small/long hairpin RNA and artificial miRNA precursors [2,11,12]. Here, we review the application of RNAi to plant disease resistance focus on: (1) approaches to induce RNAi; (2) selection of RNAi targets; and (3) pathogens targeted by RNAi.

### Approaches to induce RNAi

#### Sense or antisense viral sequences in transgene-mediated resistance

Long before the homologous sequence-dependent RNA silencing mechanism was described in 1998, virologists had discovered that transgenic plants expressing viral coat protein (CP) were resistant against infection by the homologous virus. This type of pathogen-derived resistance (PDR), termed protein-mediated resistance, has been reported in diverse viruses including tobamo-, potex-, cucumo-, tobra-, Carla-, poty-, and alfalfa mosaic virus groups as well as the luteovirus group [3,13-16]. Since then, other viral proteins also have been used to engineer virus resistance, including movement protein [17], replication-associated protein [18,19], the potyvirus nuclear inclusion proteins (NIa and NIb) [20], viral suppressor of RNA silencing [21-23], and some other viral proteins [23,24].

Initially, it was believed that viral proteins expressed from the transgenes conferred resistance [3,22]. However, unexpectedly, subsequent studies found that plants expressing the truncated viral protein sense sequence or the non-coding viral sense sequence, such as the satellite RNA sequence [22,25], also showed disease resistance to some extent. Furthermore, plants expressing antisense viral sequences also conferred high resistance. These results imply that the RNA sequence itself, in addition to the intact viral protein, participates in resistance, suggesting that novel mechanisms are involved in what is now called sense transgene-induced PTGS (S-PTGS) [5]. S-PTGS has been well-documented in viral sequence-mediated resistance. In S-PTGS, plant hosts recognize and amplify the exogenous aberrant transgenic sequence, by plant-encoding RNA-dependent RNA polymerase (RDR), into dsRNA, which serves as the substrate to trigger RNA silencing [26,27]. The resulting siRNAs can target the homologous pathogen genome for degradation. Currently, the involvement of several RDR proteins in plant defense mechanisms has been confirmed [26,27].

#### Virus-derived hpRNA transgene-mediated resistance

**Figure 1:**
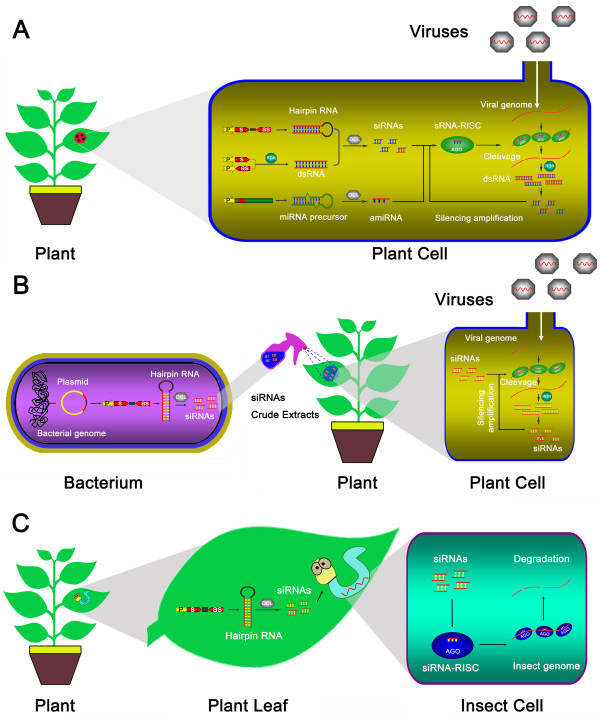
**Approaches of the application of RNA silencing to plant disease resistance.** (**A**) Expression of viral small RNA in host plants triggers antiviral silencing. (**B**) Sprayed bacterium-processed siRNAs confers resistance against virus. (**C**) Feeding on transgenic plants that carry RNAi constructs confers resistance against insect. As,antisense; P, promoter; s, sense.

Transgene RNA silencing-mediated resistance is a process that is highly associated with the accumulation of viral transgene-derived siRNAs. One of the drawbacks of the sense/antisense transgene approach is that the resistance is unstable, and the mechanism often results in delayed resistance or low efficacy. This may be due to the low accumulations of transgene-derived siRNA in S-PTGS. Moreover, numerous viruses, including potyviruses, cucumoviruses, and tobamoviruses, are able to counteract these mechanisms by inhibiting this type of PTGS [7,28]. Therefore, the abundant expression of the dsRNA to trigger efficient RNA silencing becomes crucial for effective resistance. To achieve resistance, inverse repeat sequences from viral genomes were widely used to form hairpin dsRNA *in vivo*, including small hairpin RNA (shRNA), self-complementary hpRNA, and intron-spliced hpRNA. Among these methods, self-complementary hairpin RNAs separated by an intron likely elicit PTGS with the highest efficiency [29,30]. The presence of inverted repeats of dsRNA-induced PTGS (IR-PTGS) [5] in plants also showed high resistance against viruses [31,32]. IR-PTGS is not required for the formation of dsRNA for the processing of primary siRNAs, but the plant RDRs are responsible for the generation of secondary siRNAs derived from non-transgene viral genome (Figure 1A), which further intensify the efficacy of RNA silencing induced by hpRNA, a process named RNA silencing transitivity [33,34].

The virus-derive hpRNA transgene strategy can confer high resistance in most cases; however, exceptions have been described. The resistance efficacy can vary greatly: the recovery from infection, delayed infection, or low resistance [35-37]. Although the mechanisms were unclear, several factors were believed to be involved in RNA silencing-mediated resistance. Among them, the sequence similarity between the transgene sequence and the challenging virus sequence is the most important. It has been reported that homologous viruses with sequence mutation rates of over approximately 10% to 20% in comparison to the transgene source virus will overwhelm the resistance mechanism and result in infection [38]. Additionally, it is a common phenomenon for plants to be invaded by a complex of diverse pathogen sources in the field. This might explain why high resistance against a single virus conferred by hpRNA in the greenhouse often breaks down in the field. To overcome this problem, transgenic plants with multiple hpRNA constructs from different viral sources, or with a single hpRNA construct combining different viral sequence, were created. Thus, multiple viruses can be simultaneously targeted, and the resulting transgenic plants show a broader resistance with high efficacy [1,24].

In addition to the sequence similarity, the length of the transgene sequence also contributes to high resistance. In general, an average length of 100 to 800 nt of transgene sequence will confer effective resistance [39,40]. Moreover, the efficacy is also associated with the number of transgenic copies. Additionally, hpRNA derived from multiple loci often confers effective resistance [37].

#### Artificial microRNA-mediated resistance

By mimicking the intact secondary structure of endogenous miRNA precursors (Figure 1A), artificial miRNAs (amiRNAs) are designed and processed *in vivo* to target genes of interest. The strategy of expressing amiRNAs was first adopted to knock out/down endogenous genes for functional analysis [41]. The technology is widely used in engineering antiviral plants and animals [42-48]. Compared to conventional RNAi strategies, amiRNAs have many advantages: (1) Owing to the short sequence of amiRNAs, a long viral cDNA fragment is not required; thus, the full extent of off-target effects are avoided, and the biosafety of transgenic crops is increased compared to siRNAs from long hairpin RNA; (2) Tissue- or cell-specific knock out/downs of genes of interest can be realized because of different tissue- or cell-specific promoters being used; (3) The relaxed demand on sequence length makes amiRNAs especially useful in targeting a class of conserved genes with high sequence similarities, like tandem arrayed genes, because a short conserved sequence is more easily found in these genes.

#### Transient RNA silencing-mediated resistance

Although transgenic plants created for RNA silencing exhibit effective resistance to diverse viruses, the issue of biosafety is a growing concern. To overcome this problem, a transient RNA silencing system was developed in plants by directly delivering RNA silencing molecules into plant tissues. This strategy was first tested by the mechanical inoculation of *in vitro* synthesized dsRNA triggers or the *Agrobacterium*-mediated transient expression of dsRNA in plants, and effective resistance to sequence-homologous viruses was obtained [49]. However, the high cost and considerable labor make this approach unsustainable in the field. A cost-effective approach was subsequently designed that utilized a bacterial system to biosynthesize dsRNA *in vivo*, and crude extracts were inoculated into plants via spraying (Figure 1B) [50,51]. However, in contrast to the heritable resistance mediated by transgene RNA silencing, the transient approach did not confer long-term protection [52]. Therefore, continuous spraying is required for the maintenance of protection.

### Selection of RNAi targets

#### Targeting viral silencing repressors (VSRs)

RNA silencing functions as a natural immunity mechanism in plant defense against pathogen invasion [6], and many viruses have evolved to express VSR proteins to counter host antiviral RNA silencing [53]. Mutant viruses abolishing VSR expression often display mild or no symptoms in host plants, prompting virologists to use VSR as a target of engineering resistance in plants. The first attempt, by Niu *et al.* (2006), was to express amiRNAs (based on an *A. thaliana* miR159 precursor) targeting the sequence of two VSRs, P69 of the turnip yellow mosaic virus (TYMV) and HC-Pro of the turnip mosaic virus (TuMV), in Arabidopsis. As expected, transgenic plants expressing these two amiRNAs displayed specific resistance to TYMV and TuMV [42], indicating that the strategy was applicable in engineering antiviral plants. Subsequently, transgenic *N. tobacum* expressing an amiRNA (based on an *A. thaliana* miR171 precursor) targeting another VSR, 2b of cucumber mosaic virus (CMV), also conferred resistance [43]. Recently, similar resistance was also achieved in *N. tobacum* expressing amiRNAs (based on an *A. thaliana* miR159a, miR167b and miR171a precursors) targeting TGBp1 ⁄ p25 of PVX [47].

Although VSR-targeting amiRNA-mediated RNA silencing could confer high resistance, the resistance efficacies varied greatly. In Qu’s study (2007), transgenic tobacco plants expressing an amiRNA targeting CMV 2b showed varying degrees of responses to CMV infection, including ‘resistant’, ‘recovery’, ‘delayed infection’, and ‘susceptible’ [43]. Several factors may account for these results. The first is target accessibility. The amiRNA target site might not be the optimal RISC-accessible site because not all siRNAs against a given mRNA target are equally effective. Positional effects and local secondary structures in the viral genome may block RISC access to the target site. In fact, *in vitro* RISC-mediated cleavage assays in animal systems indicated that the accessibility of RNA target sites correlates directly with RNA cleavage efficiency [11,54]. Second, natural mutation is a common strategy that viruses use to escape from host resistance under selective pressure. This was confirmed by a study of PPV chimeras with different miRNA target sequences (miR171, miR167, and miR159). Simon-Mateo *et al.* (2006) found that these PPV chimeras impaired infectivity compared to those carrying non-miRNA target sequences. Sequence analysis of the viral progeny of plants infected with these PPV chimeras showed that PPV can readily escape the miRNA-targeting pressure via mutations in the inserted foreign sequence [55]. A similar phenomenon was observed by Lin *et al.* (2009) in a study investigating the evolutionary stability of amiRNA-mediated resistance. They found that the amiRNA-mediated resistance broke down due to spontaneous or artificial mutations in the 21-nt amiRNA target sequence [56]. Third, the presence of multiple genomes leads to the replication of the non-target genome. Many plant RNA viruses contain multiple genomes. For example, CMV contains three genomic RNAs and two subgenomic RNAs. When transgenic plants expressing an amiRNA targeting the VSR are challenged by such viruses, host RNA machinery processes replicate non-target viral RNAs into virus-derived siRNAs (vsiRNA), which saturate siRISC and dilute the concentration of amiRNA-RISC to a great degree. Considering the concentration dependence of silencing, the resistance efficacy resulting from amiRNA-mediated inhibition of the VSR will be impaired or attenuated [36,44].

#### Targeting RISC cleavage hotspots in regions with conserved functions

In a previous study, to overcome the attenuation of resistance caused by the above factors, we chose the 3’UTR of CMV, which is functionally essential for CMV replication and conserved among different strains, as the target region. We searched for RISC-accessible cleavage hotspots in this region via molecular biology methods with DCL mutants, designed amiRNAs accordingly and expressed them in different host plants [44]. Most of the transgenic Arabidopsis and tobacco plants expressing amiRNAs targeting RISC-accessible hotspots, but not RISC-inaccessible spots, showed high resistance against two different strains of CMV (Shandong and Q strains). This indicated that amiRNA targeting of conserved RISC-accessible hotspots could confer higher and broader spectrum resistance than merely targeting the VSR sequence in RNA viruses with multiple genomes.

Collectively, the efficacy of amiRNA-mediated resistance is correlated with a series of elements. In addition to the 21-nt amiRNA sequence itself, the different miRNA backbones, which determine the expression level of amiRNA, and the position effects of the 21-nt amiRNA and complementary target also affect the strength of amiRNA-mediated resistance. Inconsistent with the observations in an analysis of animal viruses (poliovirus, hepatitis C virus, and human immunodeficiency virus) with mismatched target sites escaping miRNA-/synthetic siRNA-mediated RNA interference, in which mutations at either side of the central region are critical for target recognition [57-59], only the 5’ region of miRNAs is critical for the initial target RNA binding in plants [60-62]. This was confirmed by two studies using PVX chimera with miRNA target sites [55] and amiRNA targeting TuMV [56]. Using an *in vivo* assay to mutate critical positions on the 21-nt target sequence by RISC-amiRNA-mediated cleavage, three different groups were classified according to the sensitivity of resistance breakdown to position mutations, critical (positions 3–6, 9, and 12) [44], moderately critical (positions 2, 10, 11, 13, 15, and 18), and non-critical (the remaining). Furthermore, Lin *et al.* (2009) also found that the amiRNA-mediated specific resistance could be overcome by up to two mutations on critical positions within the 21-nt sequence [56]. These results support that the 5’ region and the central position of miRNAs are each critical for the initial target RNA binding and RISC-mediated targets cleavage in plants. This small RNA asymmetry should be considered in amiRNA designing for a higher silencing efficiency.

In summary, to achieve highly efficient amiRNA-mediated resistance, several factors must be considered. First, a less structured flanking region around the amiRNA target site should be chosen, providing accessibility for RISC. Second, a functionally conserved region must be used. This avoids the off-target effects caused by frequent spontaneous mutations in viral genomes. Third, the polymer strategy should be used to express more than one type of amiRNA against different target RNAs to confer resistance to viruses, as previously reported [42]. This is important because mixed infection is common. In addition, appropriate miRNA backbones should be chosen according to the specific purpose, and the amiRNA sequence itself should be assessed based on the target probably by less structured regions [44]. If all of these factors are considered, highly efficient resistance can be expected.

### Pathogens targeted by RNAi

#### RNA silencing-mediated resistance against RNA, DNA viruses, and viroids

Currently, most of the successful resistance mediated by RNA silencing has been reported against RNA viruses as most being described on the above ‘approaches to induce RNAi’ section.

In comparison to successful resistance against RNA viruses, effective resistance against DNA viruses has been rarely obtained. DNA viruses, such as geminiviruses, a family of plant DNA viruses that possess a circular and single-stranded DNA genome, seem less susceptible to RNA silencing. Fortunately, Seemanpillai reported that the expression of a transgene driven by a geminiviral promoter could be silenced by infection with the homologous genimivirus. This process has been correlated with another RNA silencing mechanism, TGS or the RdDM pathway [63], implying that the geminivirus genome may also be targeted by an RNA silencing mechanism. In fact, inoculation of blackgram (*Vigna mungo*) leaves, via bombardment with a hpRNA construct containing the promoter sequence of geminivirus *Vigna mungo* yellow mosaic virus (VMYMV) under the control of the 35 S promoter, showed that most of the plants completely recovered from the VMYMV infection [64], suggesting that the RNA silencing strategy is also effective in engineering resistance to DNA viruses.

Interestingly, a recent report showed that the geminivirus Bean golden mosaic virus (BGMV) can also be suppressed by the expression of a hpRNA transgene derived from a replicase coding sequence (AC1) [65], suggesting that a geminivirus can be targeted by both PTGS and TGS mechanisms [66-68].

A viroid is one type of plant RNA pathogen with a highly structured circular ssRNA, which does not encode any proteins and is dependent on host proteins for replication. This stable structure serves as the dsRNA substrate for the host Dicer-like enzyme [12]. As expected, it was found that abundant siRNAs were detected in viroid-infected host plants in previous studies [11,69-71]. The secondary structure was shown to restrict the accessibility of the siRNA-RISC complex, which makes viroids resistant to siRNA-RISC cleavage [72]. While the PSTVd-derived hpRNA transgene displayed wild-type symptoms similar to viroid infection in one report [73], another showed that the PSTVd genome can be targeted for degradation by the transgenic expression of a PSTVd-derived hpRNA, and this resistance is associated with a high accumulation of hpRNA-siRNAs [74], suggesting that this RNA silencing strategy may be applicable to engineer resistance to viroid pathogens.

#### RNA silencing-mediated resistance to plant fungal pathogens

Unlike plant viral pathogens, which replicate and propagate inside of the infected plant cells, interactions between some plant fungal pathogens and their corresponding host occurs via a highly specialized cell called a haustorium, which is surrounded by the extrahaustorial matrix bordered by plant and fungal membranes on either side. This represents the interface for signal exchange as well as nutrient uptake [75]. This close contact of the interaction partners might also facilitate the uptake of dsRNA or siRNA from the host plant cells into the fungal pathogens to create RNA silencing-mediated resistance. Proof of concept for this host-induced gene silencing (HIGS) of fungal genes was recently obtained for the barley powdery mildew *Blumeria graminis*, a biotrophic fungal pathogen [76]. Via transgenic expression of the dsRNA directed against *B. graminis* target transcripts in barley, a significant reduction of disease symptoms of a *B. graminis* infection was observed, whereas transgenic control that had lost the hairpin RNAi cassette was as susceptible as wild-type control plants [76], suggesting trafficking of dsRNA or siRNA from host plants into *B. graminis.* This may lead to an RNA silencing-based crop protection strategy against fungal pathogens.

#### RNA silencing-mediated resistance to plant nematodes and insects

Previous studies report several trials of directly injecting or orally administering exogenous dsRNA into insects to reduce target genes expression [77-79] and the reduced development of rootknot nematodes, as well as *Lepidoptera* and *Coleoptera* insects, feeding on transgenic plants that carry RNAi constructs against target genes in these pests [80,81]. The uptake of dsRNA or siRNA into these animals occurs by sucking or chewing on plant material, followed by resorption in the (mid) gut system, which may make this method a lasting and cost-effective method of RNA silencing-mediated resistance to insects (Figure 1C). A successful example of the control of an insect pest in the field via the RNA silencing-mediated transgenic method is targeting of the cotton bollworm gene, CYP6AE14. This gene is highly expressed in the midgut and is responsible for larval growth and cotton bollworm tolerance to cotton gossypol. The larval growth was obviously retarded, and the CYP6AE14 transcript was reduced in the midgut when cotton bollworm larvae were fed with CYP6AE14-derived hpRNA transgenic cotton, indicating that siRNAs expressed by CYP6AE14-hpRNA are active in triggering PTGS-mediated cleavage in the pest body [82]. However, we do not know whether the siRNAs are processed by plant RNA silencing machinery before spreading into midgut cells or if they are processed directly by pest RNA silencing machinery in midgut cells. Undoubtedly, the uptake of either dsRNA or siRNA or both implied that HIGS-mediated resistance might be a general approach to the application of pest control.

## Conclusions

Since the first successful application of PDR in creating virus-resistant plants, a number of strategies have been developed based on the mechanism. A better understanding of RNA silencing pathways has also contributed to the development of this technique. The RNA silencing-mediated approach is now a powerful tool in antiviral research. HIGS-mediated anti-fungal and anti-insect pathogens are also being developed. Although RNA silencing has been successful, there are still many limitations in utilizing this strategy. RNA silencing-mediated resistance and the silencing efficacy are the results of interaction between many factors, including sequence similarity, target selection, pathogen titer, and environmental temperature [83]. Thus, it is difficult to accurately predict the resistance efficacy. Moreover, to our knowledge, most of the successful examples were obtained in greenhouses. Considering that mixed infections are common in nature, it is still a challenge to obtain resistant plants. Therefore, further scientific research is required to uncover the factors affecting RNA silencing-mediated resistance in specific cases and to test the resistance efficacy in the field.

## Abbreviations

3’UTR: 3’ Untranslated region; AGO: Argonaute; BGMV: Bean golden mosaic virus; CMV: Cucumber mosaic virus; Dpi: Days post inoculation; HIGS: Host-induced gene silencing; PMMoV: Pepper mild mottle virus; PPV: Plum pox virus; PVX: Potato virus X; RISC: RNA-induced silencing complex; TGS: Transcriptional gene silencing; TMV: Tomato mosaic virus; TuMV: Turnip mosaic virus; TYMV: Turnip yellow mosaic virus; VMYMV: Vigna mungo yellow mosaic virus; Wpi: Weeks post inoculation.

## Competing interests

The authors declare that they have no competing interests.

## Authors’ contributions

CGD, CHW, and HSG drafted the manuscript. CGD, CHW, and HSG read and approved the final manuscript.
